# Existential security is a necessary condition for continued breastfeeding despite severe initial difficulties: a lifeworld hermeneutical study

**DOI:** 10.1186/s13006-015-0042-9

**Published:** 2015-05-05

**Authors:** Lina Palmér, Gunilla Carlsson, David Brunt, Maria Nyström

**Affiliations:** School of Health Sciences, University of Borås, 501 90 Borås, Sweden; School of Health and Caring Sciences, Linnaeus University, 351 95 Växjö, Sweden

**Keywords:** Breastfeeding, Caring science, Early breastfeeding cessation, Hermeneutic, Initial breastfeeding difficulties, Lifeworld

## Abstract

**Background:**

The majority of new mothers in Sweden initiate breastfeeding and many experience initial difficulties. This experience is an important cause of early breastfeeding cessation. To increase understanding, there is a need to explore the lived experiences of the decision to continue or cease breastfeeding. The aim of this study is therefore to explain and understand how this decision is influenced by the meaning of severe initial difficulties.

**Methods:**

A lifeworld hermeneutical approach was used for the study. The study was conducted in Sweden with eight mothers who experienced severe difficulties with initial breastfeeding. All except one were interviewed on two different occasions resulting in fifteen interviews. The interviews were conducted between 2010 and 2013.

**Results:**

Mothers who experience severe difficulties with initial breastfeeding feel both overtaken and violated not only by their own infants and their own bodies but also by their anger, expectations, loneliness and care from health professionals. These feelings of being overtaken and invaded provoke an existential crisis and place mothers at a turning point in which these feelings are compared and put in relation to one another in the negotiation of the decision to continue or cease breastfeeding. This decision thus depends on the possibility of feeling secure with the breastfeeding relationship. If insecurity dominates, this can, in severe cases, create a feeling of fear of breastfeeding that is so great that there is no alternative but to stop breastfeeding.

**Conclusions:**

Existential security in the breastfeeding relationship seems to be an underlying factor for confidence and therefore a necessary condition for continued breastfeeding when having severe initial breastfeeding difficulties. Unresolved feelings of insecurity may be a serious barrier to further breastfeeding that can result in a fear of breastfeeding. Such fear can force the mother to cease breastfeeding. This study highlights how women are situated in a complex cultural and biological context of breastfeeding that has existential consequences for them. An existential crisis forces mothers into a turning point for the breastfeeding decision. In the existential crisis, mothers’ responsibility for the mother-infant relationship guides continuing or ceasing breastfeeding.

## Background

Breastfeeding is considered to be a key element of public health and therefore the World Health Organization promotes exclusive breastfeeding until infants are six months old [1,2]. In Sweden nearly all women initiate breastfeeding, yet by one week after birth almost 20% and by two months after birth almost 35% have ceased breastfeeding [3].

Almost 30% of mothers initiating breastfeeding experience some kind of difficulty [4] and is a major reason for breastfeeding cessation [4-6]. Breastfeeding success is often taken for granted during pregnancy [7-9] and difficulties are thus largely unexpected by mothers [10,11]. Such idealistic expectations evoke emotional distress when difficulties occur [11,12]. Severe initial breastfeeding difficulties can lead the mother to feel lost in her role as a mother, leading to a constant struggle both emotionally and practically [13]. This struggle can be understood as a “path of determination” in which the risk is leaving the path and stopping breastfeeding [14]. Rarely, breastfeeding difficulties can evoke an existential vulnerability, forcing the mother to continue breastfeeding despite severe difficulties as she hopes to be confirmed by others as a good mother. Such vulnerability can become evident as anger towards the infant and can have negative consequences for the mother-infant relationship [15]. Physical pain and discomfort can also affect mother-infant relations [16]. Idealized expectations can clash with early problems leading to gradual disillusionment and the cessation of breastfeeding [12]. The cessation decision thus occurs in an individual, situated, and embodied context [17] wherein early cessation evokes both guilt and shame [12,18,19]. The medicalization of breastfeeding that dominates the culture in postnatal wards also has the potential to undermine women’s breastfeeding confidence [20], placing the mother in a vulnerable position in which she is not always supported by those around her [21]. This indicates a tension between the lived, embodied experience of struggling with breastfeeding and its cultural construction as natural and easy, which can threaten mothers’ emerging maternal identities [22].

Additional factors may lead to early cessation. Examples of *biological factors* are painful breasts, infants’ suckling difficulties [23-25], low milk supply, perceptions of low milk supply and perceptions of infants as not satisfied with breast milk alone [5,23,24]. Other concerns related to infant and maternal health such as illness and the need for certain medications can be reasons why mothers stop breastfeeding [5]. Birth by cesarean section is associated with more breastfeeding problems than vaginal delivery [24,26] as well as being associated with more postpartum pain [27]. Among primiparae a high BMI is associated with a risk of early cessation of exclusive breastfeeding [28]. Examples of *factors related to care* and care routines can be the separation of mother and infant before the first breastfeeding session [29] and supplementation with formula [30]. Regulating and disempowering attitudes [31] as well as limited knowledge about breastfeeding among professional carers [32] are barriers to breastfeeding. *Psychological factors* that influence breastfeeding negatively can be the mother’s intentions, low levels of confidence, and having low self-efficacy [33,34]. The mother’s lack of knowledge about breastfeeding [35] and lack of support [36-39] are other psychological factors. *Sociodemographic factors* such as being a first-time mother or a smoker have also been described as negative factors [40,41] along with being a young mother [24].

The findings presented above indicate that the breastfeeding decision and outcome are complex issues influenced by diverse circumstances [17,42]. As many mothers experience initial difficulties leading to early cessation [4] it is necessary to further understand the complexity of the breastfeeding question for these mothers, particularly in a Swedish context with a high level of women initiating breastfeeding but also a relatively high level of early cessation.

This was the point of departure for a research project focused on the initiation of breastfeeding. The project started with two phenomenological studies describing women’s lived experiences of initiating breastfeeding, one with mothers experiencing well-functioning breastfeeding [43] and another with mothers experiencing severe initial difficulties [13]. The results from these studies prompted questions about the breastfeeding decision and why some mothers continue breastfeeding while others cease breastfeeding when having severe initial difficulties. A case study was then conducted which highlighted that existential vulnerability can be evoked, forcing a mother to continue breastfeeding despite having severe difficulties [15]. But one question remained: why do mothers continue *or* cease breastfeeding when having severe initial difficulties? In order to answer this question, this study aims to explain and understand how women’s decisions to continue or cease breastfeeding are influenced by the meaning of severe initial difficulties.

## Methods

A lifeworld hermeneutical approach was used in order to explain and understand the breastfeeding decision in the context of severe initial difficulties [44]. Hermeneutics is the philosophy of understanding gained through interpretation and forms an important part of the basis of human sciences [45]. There are several different hermeneutical approaches and among them is the lifeworld hermeneutical approach. Lifeworld refers to Husserl’s epistemological idea to go “to the things themselves” in other words, it entails researching a phenomenon. The question of meaning is central to this form of research [44].

Openness, such as having an open attitude to the research phenomenon is necessary for meaningful and valid proposals for how the investigated phenomenon can be explained and understood. The first inspiration for this empirical approach is the work of Gadamer [46]. His hermeneutical philosophy can be summarized as efforts pursuing an open approach by encouraging awareness and a critical attitude to one’s history and pre-understanding. According to Gadamer, this is necessary for scientific consciousness and to shed light on “the otherness” of the phenomenon [46]. In order be able to suggest how to explain latent meanings in the data, a second source of inspiration was found in Ricoeur’s hermeneutical approach [47]. Gadamer shows us the importance of openness and suspicion towards ourselves, while Ricoeur, on the other hand, provides us with the notion of being suspicious towards the text and its “surplus of meaning” (ibid) in order to interpret latent meanings. It is when the open approach suggested by Gadamer limits understanding that Ricoeur emphasizes the need to go beyond the given conditions in the data in order to reach interpretations that are meaningful. Ricouer’s proposal to include explanations for understanding a phenomenon has therefore also guided the analysis in this study.

### Participants and data collection

The study was conducted in Sweden using lifeworld interviews with eight mothers who experienced severe difficulties with initial breastfeeding. A lifeworld interview is phenomenon-oriented, focusing on the meanings of the lived experiences [44].

Participants were therefore chosen because of their varied lived experiences of initial breastfeeding difficulties. Swedish-speaking mothers to healthy, singleton, full-term infants who were experiencing initial breastfeeding as very difficult were invited to participate by a midwife in the maternity ward or in the breastfeeding clinic co-located with the maternity ward. All the women who were asked to participate gave their consent. In total, seven mothers were primiparae and one was multiparous with an age range of 20–37 years. The range of difficulties included breast complications, infections and abscesses, infants with problems suckling and/or other difficulties. As a result of these breastfeeding problems, the mothers required prolonged maternity stays, breastfeeding clinic care and/or support from other care settings. These circumstances were also criteria for inclusion. Participants’ birth experiences were normal, traumatic or complicated (e.g. cesarean section and sphincter rupture). All lived with the father of the infant in rural or urban areas. At the time of the first interview, four women were breastfeeding exclusively, one was exclusively pumping, and three had ceased breastfeeding. The duration of breastfeeding was between two weeks and one year.

All interviews were conducted between 2010 and 2013, audio-recorded and transcribed verbatim by the first author (LP).

All mothers except one were interviewed on two different occasions resulting in fifteen interviews. The first interview occurred within two months of birth and took place in the women’s homes. The initial interview question was, “Would you like to tell me about your experiences initiating breastfeeding?” In order to obtain in-depth reflection and encourage the interviewees to describe variations in their experiences, questions such as, “Can you tell me more about that?” and “What does that mean to you?” were asked during the interview. The interviews were guided by openness and pliability to the women’s experiences and therefore no interview guide was used.

For the follow-up interview, the first author (LP) asked the mothers to participate again and all of them agreed except one who could not be contacted. These interviews were carried out between one-and-a-half to three years after the birth. These follow-up interviews were based on the meanings that evolved from the first interview with each interviewee. The open and reflective attitude focusing on meanings was also applied to this follow-up interview.

### Data analysis

Analysis was conducted using a lifeworld hermeneutical approach developed for empirical hermeneutics by Dahlberg, Dahlberg and Nyström [44] with inspiration from Gadamer [46] and Ricoeur [47]. The analysis thus consisted of interpretations made in order to explain and understand the phenomenon in focus.

The analysis process began with multiple readings of the first interview transcripts to gain an overview of the women’s experiences. This was followed by a shift in attention to the various emerging meanings of the phenomenon, therefore moving between the whole (the first interviews) and parts (interpretations of statements) practicing openness and reflection on the data [44]. The meanings were sorted, grouped and interpreted into six themes, each suggesting explanations for an understanding of mothers’ breastfeeding decisions. Each theme was analyzed and interpreted as *thesis-antithesis-synthesis*. The *thesis* consisted of interpreted meanings related to the decision to continue breastfeeding and the *antithesis* consisted of interpreted meanings related to the decision to stop breastfeeding. *Thesis* and *antithesis* were compared and interpreted formulating a *synthesis*. In this phase, a search for similarities and differences in meanings was carried out in order to explain and understand in a preliminary way patterns of meaning.

After the follow-up interviews, the six preliminary interpretation themes described above were re-evaluated. During this evaluation the researcher again moved, with an open and reflected mind, between the whole (the first and the follow-up interviews) and parts (interpretations of statements) in order to evaluate the themes. During the entire analysis process, all interpretations were validated by attempting to falsify them against data by searching for contradictions. Interpretations were considered valid if they were consistent with the whole and the parts. During these checks, some preliminary interpretations were further developed while others were reworded or rejected because they did not fulfill the validity criteria [48].

During the analysis, the interpretations were not only directed towards meanings but also to why those meanings appeared, including intentional explanations [44]. At this stage, Ricouer’s hermeneutical ideas in which explanations are included in the proposals for new understanding [47] influenced this analysis which was performed by moving beyond given conditions, thus avoiding causal explanations but also proposing intentional explanations, i.e. explanations of the background to the suggested meanings. An intentional explanation can for example, highlight latent meaning about how a person understands her situation and what it means to be in that situation. Quotations from the interviews illustrate the six interpreted themes and the participants cited are identified numerically (ID01 to ID08).

Finally, a further comparative analysis among the six interpreted themes gave rise to the main interpretation that linked all the previous interpretations together in the form of *thesis-antithesis-synthesis*. For the main interpretation, the parts were reviewed against the whole and vice versa. The suggested interpretations concerning each theme thus created parts of a new whole embracing all previous interpretations. The main interpretation thus linked together all the different interpreted meanings.

### Ethical considerations

Approval and permission to undertake the study were obtained from the Ethics Committee of the Medical Faculty at the University of Gothenburg, recorded (Dnr 373–12). All participants received written and verbal information and written consent was given by each informant. Due to the sensitive nature of the topic, arrangements were made for all women to have access to an extra appointment with a professional caregiver at the breastfeeding clinic, however this was not needed.

## Results

The findings are presented as six interpreted themes, each consisting of thesis and antithesis followed by synthesis. This structure is outlined in the first theme. The themes are followed by a comprehensive understanding in a main interpretation that suggests how to explain and understand the phenomenon in focus.

## The meaning of the infant

### Thesis

When the mother receives positive responses from the infant in the breastfeeding relationship, she feels confirmed as capable of caring for the infant. In that moment, she interprets that the infant is doing well, which motivates her to continue breastfeeding.

When he was that sad and I understood that he wanted to eat and I tried that and when it works well then it’s strengthening [for me]. This feeling is beginning to come now // yes, it’s strengthening me. I just feel more confirmed (ID03).

### Antithesis

When the mother lacks a positive response, she does not feel confirmed, and that decreases motivation for breastfeeding. She cannot then see that the infant is doing well and she becomes afraid of hurting the infant.

Those feelings when he didn’t take the breast were probably the most important for me stopping. I felt frustrated and powerless. I tried, changed [breastfeeding] positions and tried everything I could and yet it still was not working. It was terrible to feel . . . that the infant wasn’t doing well (ID04).

### Synthesis

***The mother’s perceptions of the infant guide her breastfeeding decisions*** and her experience of herself in the breastfeeding relationship. Her own understanding of the infant’s response will determine whether she feels confirmed as a breastfeeding mother or not. When the mother experiences confirmation, this leads to her feeling capable of caring for the infant. The infant then confirms not only the mother’s capability, but his or her own wellbeing. When the mother, on the other hand, does not feel confirmed, she loses faith in herself as a breastfeeding mother. She concludes that the infant is mistreated by breastfeeding and her motivation to continue breastfeeding is lost.

### The meaning of the body

The experience of positive responses from the body such as a good milk supply or less painful breasts means confirmation of the body’s capability of breastfeeding. This creates confidence in the body’s breastfeeding ability and leads to continued breastfeeding.

Then when he had gained weight, 500 g in a week, it was quite a relief // But before that, it was hard because for breastfeeding to work out well it was dependent on me and my body. And I didn’t know if there was something wrong with me or if there was enough milk (ID02).

I got confirmation that there actually was milk there, then I was calm (ID06).

When the mother, on the other hand, feels that the body largely generates negative responses, this means a lack of confirmation, which in turn raises doubts over the body’s function. Feeling that one’s own body hurts too much creates both physical and emotional suffering that make breastfeeding actually impossible.

It was terrible . . . I couldn’t have a sweater on, I couldn’t go outside, I had to pull down every blind, it was like a dark prison and then just focusing on breastfeeding that wasn’t working and it did not get any better when I had an infection and an abscess. If there had been any improvement, but there wasn’t. I was terribly depressed (ID08).

But nothing came. Nothing came out of the breasts. It was . . . no . . . nothing (ID01).

***The body provides hope or mistrust depending on its responses*** crucial for the breastfeeding decision. This contributes to a presence or absence of confirmation of the body’s ability to breastfeed. When the body gives positive responses, such as a good milk supply or less painful breasts, it provides hope and confidence in the body’s ability, which becomes a positive sign. A lack of positive signals from the body contributes to a sense of being trapped in the body, making the mother mistrust its function. The feeling that the body desires to be released from suffering arises and the situation is so painful that breastfeeding becomes unbearable.

### The meaning of anger

Breastfeeding difficulties may give rise to anger that is directed outwards towards a person or towards the situation in a more or less conscious attempt to find external explanations for the difficulties. Anger can be directed towards, for example, the infant, the health care received, and others’ expectations. This may then be a reason to stop breastfeeding; paradoxically, however, it can also be a motivator to continue.

I felt that . . . I hate this [breastfeeding relationship and the expectations about breastfeeding] . . . then I called my mother . . . and I said I hate to breastfeed, then I had a real breakdown . . . But then she said “Then you shouldn’t breastfeed if you hate it” . . . and then we talked about it for a while and then I felt that I had some strength to continue (ID03).

On the other hand, when the anger results in breastfeeding cessation, it eases mothers’ feelings of responsibility and protects mothers from feelings of extreme failure.

I think that those working on the maternity ward destroyed my breastfeeding // I think they were worthless. Didn’t take any consideration or none of them asked me anything about my wellbeing // It made me so damn angry (ID01).

***Anger facilitates handing over responsibility to others***. Anger makes it possible to attribute to others the responsibility for difficulties and this protects the mother from bearing all the responsibility herself. The anger can thus be understood as a release related to attempting to find explanations outside of the mother herself. The anger toward others can make her feel both stronger, as protection from self-loathing, or it can drain her inner strength. It is nevertheless how anger is handled and to what it is attributed that determines how such protection works. If the mother has the opportunity to be angry and then the ability to move on, anger can help as a motivating force for continuing breastfeeding. If the mother, on the other hand, is too overwhelmed with anger, the anger and attempts to find explanations outside of herself can paralyze her. The only solution to the risk of feeling defeated is to stop breastfeeding.

### The meaning of expectations

When breastfeeding difficulties arise, the mother feels that she is being watched both by herself and others. The expectations become obvious and the mother is ashamed to differ from the expected image. Fear over what others may think generates a need to perform as a happy mother breastfeeding her infant.

It’s that I compared myself with others. Everyone else managed it and this is how it is in general in society. Why can’t I handle it then? It isn’t about jumping 2.4 meters in the high jump // to have severe difficulties isn’t normal . . . , I felt a little strange and worthless. I wanted to breastfeed to prove my ability to do it (ID05).

Such expectations can also mean that breastfeeding is experienced as too great a performance to manage. The gaze from others reinforces the mother’s own sense of being a failure, a feeling of being an unsuccessful mother to her infant.

I have the feeling that others will think that I am bad because I cannot give him what he wants [breastfeeding]. Initially, there are a lot of emotions and I feel forced to try to breastfeed. Otherwise, others will think that I am a bad mother // But I couldn’t manage to handle the expectations and I stopped breastfeeding (ID07).

***Feeling others’ gazes functions as a gauge for the breastfeeding decision*** as well as how the mother sees herself. She thus becomes her own judge; whether breastfeeding is continued or not depends on her strategy in dealing with this dilemma. If a feeling of shame develops upon being unable to fulfill expectations, the need to eliminate this feeling forces the mother to continue breastfeeding. Continuing serves the purpose of demonstrating her ability to other people, and is therefore a driving force in continuing breastfeeding despite difficulties. The shame can, on the other hand, be perceived as so unbearable that the mother feels herself a failure. To escape the judging gaze of others, the mother reformulates and devalues the importance of breastfeeding, which facilitates breastfeeding cessation.

### The meaning of loneliness

Breastfeeding difficulties can create feelings of loneliness that risk reinforcing feelings of uselessness and difference from other mothers. In order to escape loneliness, safety is sought in others who can provide support for continued breastfeeding. The connection with others provides a sense of belonging that makes breastfeeding possible.

[My friend] had the same troubles and we talked a lot about that. And then I had some other friends who I talked to on the telephone, they have had troubles too and then I was very much at home alone during that time (ID05).

The reverse is also true. The feeling of loneliness may lead to withdrawal because of a fear of being detected as underperforming, useless, and different. Such fear constitutes a barrier to continued breastfeeding. Instead, the mother searches for contact with others who have stopped breastfeeding and thus breastfeeding can be ceased.

I didn’t talk to [the nurses] about my problems or about how I felt but I kept face // I didn’t want to cry and collapse there and then, instead I went to them to weigh and measure him and then I went back home (ID04).

***The fear of being discovered as being different from other new mothers becomes a barrier for breastfeeding*** and seems to balance against a desire to find someone to contact and create a sense of belonging with. The strength of this fear influences the direction of the search for belonging. The choice is between searching for support from people who represent a possibility for continuing breastfeeding or from people who represent breastfeeding cessation. It seems to be the need for identification with others in similar situations that determines mothers in favor of breastfeeding continuation; otherwise, the difference from other mothers becomes too obvious. If the feeling of loneliness and the fear of being discovered as different become too extensive, the mother cannot bring herself to break out of it and breastfeeding ends with a lasting feeling of being different and unsuccessful.

### The meaning of care

When the mother is able to meet professional carers who are trustworthy and extend their care beyond the biological body, a feeling of possibility to overcome breastfeeding difficulties can result. These carers facilitate the development of self-confidence, which eases and reduces thoughts of worthlessness. This is true even if these mothers have experienced non-caring behavior from some of the carers involved.

That lump in my throat or in my chest completely disappeared. Suddenly I became much more alert and all difficult thoughts that I had when I felt myself to be insufficient disappeared. She [the carer] gave me the right feeling or the right attitude for managing it (ID02).

On the other hand, the care can be experienced as non-caring as, for example, intrusive hands-on breastfeeding help, or care that focuses solely on the infant or the body. Such care is degrading in that it objectifies the woman and reduces her to solely an instrumental functionality. Although individual carers may try to disrupt this pattern, it seems to be of no importance for breastfeeding continuance. The feeling of being a worthless object has already been integrated too much in the mother’s self-concept for breastfeeding to continue.

I was extremely vulnerable, and then they tried to help me with an intrusive hands-on approach. I said “yes” to that because I didn’t know how to do it // It felt even more like I couldn’t manage it by myself and instead someone needed to help me. Instead, someone could have been sitting beside me letting me try // It felt like a violation of my own body (ID04).

***An instrumental way of giving care undermining mothers’ breastfeeding*** and seems to be based on the idea that a woman who has just given birth does not have the same need for extra care as for patients who, for example, are being treated for some medical condition. A new mother can nevertheless be exhausted, in healing from surgical procedures, and under the influence of the hormonal transition that occurs when the milk comes in. The major life change may also give rise to existential issues and sometimes even feelings of depression. Being allowed to have the same care needs as for a “real” patient appears to be significant for the mother’s possibility to overcome breastfeeding difficulties; feeling that her feelings are accepted enables the mother to feel that her burdens become lighter because they are shared. If this is possible, she feels confirmed and strengthened in continuing breastfeeding. Yet in the absence of such acceptance, suffering becomes overwhelming, leading to her feeling forced to cease breastfeeding.

### Main interpretation - existential security or insecurity determines mothers’ breastfeeding decision

Mothers who initiate breastfeeding with severe difficulties may feel overtaken and violated by the needs and demands from her infant, the extensive pain and/or changes in her body, and her own as well as others’ expectations for her to succeed. Contact with professional carers whom she experiences as too demanding or her own feelings of anger and loneliness may further enhance these feelings of being overtaken and violated. In these cases, the mother knows that she must take responsibility for the mother-infant relationship, especially given its exposure and vulnerability due to initial difficulties.

In such circumstances, the breastfeeding relationship is characterized by insecurity and complexity because of the different aspects of the feelings of being overtaken and violated, feelings that seem to be interwoven. She does not recognize herself as she was prior to giving birth, but is well aware of the advantages of her own milk as the best kind of nourishment. Implicitly, she also seems to understand that the issue of breastfeeding has an existential aspect symbolically linked to the mother-infant relationship. Consequently, she has to take responsibility for both the relationship with the infant and the infant’s nourishment in order to support the development and wellbeing of the infant.

When feelings of being overtaken and violated make her consider her body primarily as a biological tool, separated from the mother-infant relationship, feelings of alienation easily emerge. The intended reciprocal and intimate relationship with the infant becomes the opposite wherein it is difficult to feel closeness. It is when feelings of alienation make the relationship with the infant problematic that she must find a solution. She approaches an important turning point, an existential crisis, wherein it is necessary to decide whether she should continue to breastfeed or not. She negotiates with herself and tries to find a path that is advantageous for herself and her infant. The different aspects of the feelings of being overtaken and violated are weighed against each other and put in relation to the context of her responsibility for the infant’s thriving and the development of her relationship with the infant.

If it is possible to find togetherness with others who, directly or indirectly support breastfeeding, it becomes easier to continue. The capacity of others to make the mother secure in the breastfeeding relationship seems to make it possible for her to reconcile herself with her initial difficulties. Slowly but surely, she begins to feel secure with continued breastfeeding. This security helps her to regain a mutual intimacy with the infant thus enabling closeness.

The mother’s overwhelming feelings of suffering, anger and loneliness lead to a feeling of alienation from the breastfeeding relationship that can encourage her to see the decision to stop breastfeeding as an act of caring responsibility. In this case, the mother experiences herself as excluded from all other mothers who appear to be successful as well as abandoned by the health carers she has encountered, leaving her unable to continue breastfeeding without jeopardizing her closeness to the infant.

The link between feelings of security and the mother’s relationships to other people is clear. Yet it is important to keep in mind that “other people” represent a variety of contexts, such as professional caregivers, family members, friends and not least the infant. Most critical for the sense of security or insecurity is perhaps the new mother’s tendency to look at herself from the outside, imagining others’ perceptions of her and she thus becomes her own toughest critic in the decision to continue or cease breastfeeding. Others’ gazes and her own beliefs about what they see create feelings of either security or insecurity that in turn relate to the development, or not, of the fear of breastfeeding.

It thus seems fair to assume that the decision to continue or cease breastfeeding after having severe initial difficulties depends on whether or not it is possible to feel existential security in the breastfeeding relationship. Maintaining this security can thus be performed in different ways; mothers can, for example, find security through confirmation from others. The reverse is also true. If existential insecurity dominates, mistrust, doubt, frustration, and worry easily follow, and in severe cases this creates a fear of breastfeeding that is so great that there is no alternative but to cease breastfeeding.

## Discussion

This study has highlighted existential dimensions in new mothers’ decisions to continue or cease breastfeeding when faced with severe initial difficulties. This decision appears to be dependent on her sense of either security or insecurity in the breastfeeding relationship.

The result regarding the presence of feelings of security as necessary for continued breastfeeding is notable as previous research primarily highlights maternal confidence as a predictor for the breastfeeding decision. These studies indicate that mothers with high self-efficacy (confidence in breastfeeding ability) are eager to continue breastfeeding [33,49]. Additionally, the importance of a confident commitment in order to continue breastfeeding has been shown [50]. The present study thus contributes to the body of knowledge about breastfeeding, emphasizing that the existential dimension of security is a strong underlying factor for confidence in one’s own ability to breastfeed. This understanding can be further understood and bolstered by considering Bowlby [51] and Maslow [52] who recognise security as a foundational element in human relations and as a motivating factor in human needs. A study by Persson et al., has shown that mothers’ sense of security in the first postnatal week is dependent on support from staff and family as well as the capacity and health of the woman and infant, including well-functioning breastfeeding [53]. Our study concurs with these findings and further contributes by emphasizing existential security’s influence on the decision to continue or cease breastfeeding in the face of initial difficulties.

In order to feel existentially secure, the mother’s interpretation of others’ perceptions of her appears paramount. The infant’s perceptions seem most significant. A well-nourished and satisfied infant provides the mother with the confirmation necessary for feelings of security. On the other hand, a poorly nourished or dissatisfied infant generates a sense of existential insecurity that, at worst, can create a fear of breastfeeding. This insecurity can also be understood by the work of Hinsliff-Smith et al. which suggests that mothers seem unprepared for infant demands, contributing to a rollercoaster of emotions [18]. This lack of understanding about infant feeding behavior can result in the decision to stop breastfeeding. In a recent study by Larsen and Kronborg, it was suggested that giving up breastfeeding can be crucial but necessary for the infant’s health and well-being [21], which was similar to the findings in the present study in which a mother’s feelings of responsibility for her infant’s nourishment, biological but also existential, can inform the decision to stop. These indicate that mothers, in order to feel secure, need to be prepared for the behavior and demands of infants in order to interpret cues in a constructive way. Hegney et al. suggest that mothers with extraordinary breastfeeding problems come to a breaking point were a decision is made [19]; this is in also in line with our study, which describes a turning point for making the decision. According to this, it is thus important to understand that a mother’s decision to stop breastfeeding is a way to take responsibility for the relationship as well as the wellbeing of both mother and infant. Breastfeeding cessation can, in fact, be crucial for the mother in her efforts to bond with the infant and vice versa. Kelleher also notes that this is an important aspect for health care professionals to consider [16].

In severe cases, the mother’s sense of existential security has been completely eliminated leading to a fear of breastfeeding that is grounded in the exposed situation and the feeling of being alienated from both herself and her infant; this is ultimately destructive to the possibility of continued breastfeeding. The meaning of the phenomenon “fear of breastfeeding” does indeed need phenomenological and hermeneutical investigation from further empirical studies. Yet previous research contributes to tentative knowledge in this area by showing that mothers with negative breastfeeding experiences may report lower levels of self-efficacy compared to mothers with more positive experiences [33,49]. This indicates that the lived experiences are of great importance for the mother’s breastfeeding of a future infant [54]. The phenomenon “fear of breastfeeding” can be further understood using the ideas of the French philosopher Merleau-Pontys [55] regarding the lived body. According to him, the memories from experiences are etched in the body, making it reasonable to assume that memories from previous breastfeeding experiences can contribute to a troublesome breastfeeding relationship both with the present infant and a subsequent infant. It is also suitable to assume that experiences of breastfeeding difficulties may remain with and affect the mother during future breastfeeding experiences. This statement is strengthened by previous research showing that previous breastfeeding experiences can influence the desire to breastfeed again [15,54].

One way to strengthen the mother’s feeling of security is to provide care and support. Previous studies that have found individual, on-going face-to-face support [36] consisting of relationship-building [37] and characterized by authentic presence [56] is key for breastfeeding continuation. In addition, a process-oriented training in breastfeeding for health care professionals influenced women’s abilities to handle breastfeeding difficulties [57]. It is thus obvious that support is important in order to promote further breastfeeding. The results from our study imply that it can be valuable for health care professionals to be aware of existential aspects of the experience and that existential security or insecurity affects the breastfeeding decision together with the more practical and biological/medical aspects. One of the themes in this study, the meaning of care, together with the main interpretation, can guide the caring approach. We suggest, therefore, the benefits of an attitude of permission and acceptance of the individual mothers’ existential feelings from the health care professionals involved. Permission includes being allowed to be emotional and having the opportunity to talk about experiences as a way to work through them and reach a state of reconciliation. By doing this, the mother can be more aware of possible feelings of shame, loneliness and anger, which can be made comprehensible, thus lessening the feeling of alienation from herself and the infant. Similarly, Hegney et al. show the importance of trustworthy relationships with health care professionals for breastfeeding continuation [19]. Smythe et al. also emphasize that postnatal care should foster a space for dwelling [58]. On the other hand, the present study has identified barriers to such dwelling and strengthening support in postnatal care, mainly when it tends to provide care that is focused on the biological body in an instrumental way such as intrusive hands-on breastfeeding care. Such care has the potential to undermine women, leading to insecurity, fear and cessation of breastfeeding. This is not unique; Nelson [9], Dykes [20] and Schmied et al. [56] also point in that direction.

It is therefore important that health care professionals have the ability to extend their care beyond the biological body and the instrumental way of caring, all the while being aware of the potential barriers of anger, loneliness, fear of discovery and the perceived gaze from others; otherwise the risk of early cessation of breastfeeding can be realized. This extended care as described above is also important because it has been shown that birth traumas such as cesarean section or severe vaginal ruptures influence breastfeeding; notably, some of the mothers in our study experienced birth trauma. Mothers who experience birth trauma have more breastfeeding problems than mothers with normal vaginal births [24,26,27,59]. The influence of this may well be a great barrier for breastfeeding when the naturalness of childbearing is emphasized more than the needs of the postoperative patient. In such situations, it is important to be respected as a patient suffering from trauma and not solely as a healthy mother who has given birth to a healthy infant. Breastfeeding can be difficult to manage because of the pain and discomfort that accompanies this trauma. Similar conclusions can be drawn from another study [60] by Beck which shows that birth trauma has an impact on breastfeeding experiences resulting in two pathways: persevering with or curtailing breastfeeding. Physical and/or psychological trauma, such as feeling violated and stripped of dignity during childbirth and early breastfeeding, can thus have an impact on the decision to stop breastfeeding. With this in mind, new mothers, especially those with severe initial breastfeeding difficulties, need to be met in a sensitive way that allows them to reconcile themselves to their feelings of alienation and come close to their infant, regardless of continued or stopped breastfeeding.

Lastly, some question about strengths and weakness in the present study must be addressed. The strength of a lifeworld hermeneutical study is the opportunity to understand the phenomenon from the participant’s perspective. However, the relatively low number of participants can in a positivistic view, be seen as a hindrance for generalization. Lifeworld studies such as this one are phenomenon-oriented, focusing on meanings in the lived experiences: “the truth” is in these meanings. Therefore the matter of “truth” relies on the amount of depth in the descriptions of meanings rather than the specific quantity of participants or interviews [44]. This study consists of eight participants and fifteen interviews that were of sufficient depth to meet the criteria for such studies. The fact that the first interviews were performed within two months of birth and the initiation of breastfeeding enabled fruitful and rich descriptions of the experience of women’s decision-making around breastfeeding. The same was true of the interviews performed up to three years after the birth. The risk for a negative recall factor could still be a deficit, yet it was clear from the interview data that breastfeeding difficulties affect women in such a way as to make them acutely memorable; the women had no problems talking about their past experiences. Both the initial and follow-up interviews with each woman were analyzed together as equally important in relation to the meaning of the experiences and decision-making around breastfeeding. This may have mitigated the effects of women’s possibly reconsidering or working through their past experiences. The fact that the researcher and participants had previously met can also been a positive factor minimizing the potential risk of negative recall. The follow-up interviews were based on issues discussed in the initial interview which may also have made it possible to connect to and deepen the meaning of past experiences. Nevertheless, it is necessary to bear in mind that human science research such as lifeworld hermeneutics is always contextual. It is therefore important to consider the result in light of its context, i.e. mothers in Sweden having severe difficulties upon initiating breastfeeding. The result is then expressed in the form of a main interpretation, a section that intends to turn interpretation into understanding, making generalization possible. The principle of a movement between the whole (the whole set of data) to the parts (interpretations of parts) to the new whole (the main interpretation) in the analysis is central for this possibility. The main interpretation can therefore be applied in a more abstract way in other contexts than those in the study, making generalization possible [44].

## Conclusion

Existential security in the breastfeeding relationship is an underlying factor for confidence and hence a necessary condition for continued breastfeeding after having severe initial breastfeeding difficulties. Lingering feelings of existential insecurity may be a serious barrier to further breastfeeding which can result in fear of the breastfeeding relationship. Insecurity and fear of breastfeeding force mothers to stop breastfeeding. This study highlights how women are situated in a complex cultural and biological breastfeeding context entailing existential consequences. The existential crisis forces mothers into a turning point for the breastfeeding decision wherein mothers’ responsibility for the mother-infant relationship guides continuing or ceasing breastfeeding. Health care professionals need to be aware of this complex existential crisis related to security or insecurity and its effects on the breastfeeding decision when caring for mothers having initial breastfeeding difficulties.
